# Bridging the ICD11 and the DSM-5 personality disorders classification systems: The role of the PID5BF + M

**DOI:** 10.3389/fpsyt.2023.1004895

**Published:** 2023-03-02

**Authors:** Rute Pires, Joana Henriques-Calado, Ana Sousa Ferreira, João Gama Marques, Ana Ribeiro Moreira, Bernardo C. Barata, Marco Paulino, Bruno Gonçalves

**Affiliations:** ^1^Faculdade de Psicologia, Universidade de Lisboa, Alameda da Universidade, Lisbon, Portugal; ^2^CICPSI, Faculdade de Psicologia, Universidade de Lisboa, Alameda da Universidade, Lisbon, Portugal; ^3^Instituto Universitário de Lisboa - Business Research Unit (BRU-IUL), Lisbon, Portugal; ^4^Clínica Universitária de Psiquiatria e Psicologia Médica, Faculdade de Medicina, Universidade de Lisboa, Avenida Professor Egas Moniz, Lisbon, Portugal; ^5^Consulta de Esquizofrenia Resistente, Hospital Júlio de Matos, Centro Hospitalar Psiquiátrico de Lisboa, Lisbon, Portugal; ^6^Centro Hospitalar de Lisboa Ocidental, Hospital de Egas Moniz, Lisbon, Portugal; ^7^Departamento de Psiquiatria e Saúde Mental, Centro Hospitalar Barreiro Montijo, Av. Movimento das Forças Armadas, Barreiro, Portugal

**Keywords:** ICD-11 Classification of Personality Disorders, DSM-5 Alternative Model of Personality Disorders, personality disorders, severity, personality traits, LPFS-SR, PID-5, PID5BF + M

## Abstract

**Introduction:**

In both the ICD-11 Classification of Personality Disorders and the DSM-5 Alternative Model of Personality Disorders (AMPD) personality disorders (PD) are characterized by impairments in self- and interpersonal functioning which distinguish the various levels of dysfunction. Moreover, pathological traits are used by these classification systems to define the stylistic expression of personality dysfunction. Negative affectivity, detachment, antagonism/dissociality, and disinhibition feature as trait domains in each of these models. However, there are also differences between the two models, namely, in the psychoticism domain, which does not feature as a personality trait domain in the ICD-11, and in the anankastia domain, corresponding to compulsivity in the DSM-5, which was removed from the final AMPD model. Furthermore, facets are acknowledged by the DSM-5 within each trait domain, while this does not occur in the ICD-11. In view of the similarity between these classification systems, their harmonization would be beneficial for the clinical profession. With this goal in mind, the PID5BF + M, an algorithm that assesses the DSM-5 and ICD-11 six trait domains and 18 facets, was developed and has proven to adequately characterize the ICD-11 trait domains by means of DSM-5 trait facets.

**Methods:**

The current study compares a community sample (*N* = 280, *M*_*age*_ = 48.01, 53.2% females) with a PD sample (*N* = 131, *M*_*age*_ = 42.66, 45.0% females) along with the PID5BF + M, the LPFS-SR and the PID-5. Given that the PID5BF + M total can be seen as a measure of the level of personality dysfunction, strong relations between the PID5BF + M total and the LPFS-SR total are expected. Strong relations between the trait specifiers measured by the PID5BF + M and the PID-5 are also expected. Finally, the community and clinical samples are expected to differentiate by means of the dimensions assessed through the three afore-mentioned measures. The Spearman rank-order correlation coefficient was used to measure the strength and direction of associations between the PID5BF + M total and the LPFS-SR total and between the PID5BF + M and the PID-5 traits. Group differences were explored using the Mann–Whitney *U* test for independent samples.

**Results:**

As expected, there were strong, significant, and positive relations between the measures. Furthermore, higher scores were observed in all the variables for the PD group against the community group.

**Discussion:**

Although this study has limitations, its findings sustain that the PID5BF + M has potential to assess the severity of personality disfunction and to characterize the stylistic features of PD as they are conceived by both the ICD-11 and the DSM-5. Although more research is needed regarding the convergent validity of the PID5BF + M, this new test contributes to the harmonization of both systems and to parsimony in the assessment of PD, which is the main objective of clinical practice.

## Introduction

Widely acknowledged problems such as comorbidity and within-category heterogeneity have stemmed from the assumption that mental disorders are dichotomous (1–5). Given the continuous nature of psychopathological variation, as shown by the empirical evidence, both the ICD-11 Classification of Personality Disorders (6) and the DSM-5 Alternative Model of Personality Disorders (AMPD) (7) proposed dimensional models to classify PD. Thus, personality assessment and PD diagnosis comprehend an evaluation of the level of personality functioning, the central PD feature (8–11), and of specific personality traits that mirror the severity of the dysfunction.

Regarding impairments in self- and interpersonal functioning, both the ICD-11 and the DSM-5 delineate levels of dysfunction. In the ICD-11 the clinician assesses the degree of personality dysfunction considering three levels of severity (mild, moderate, and severe), described in the ICD-11 Clinical Descriptions and Diagnostic Guidelines for Personality Disorders (6). The DSM-5 proposes the Level of Personality Functioning Scale (LPFS) (7, 11) that differentiates five levels of functioning, ranging from healthy/adaptative functioning (Level 0) to some (Level 1), moderate (Level 2), severe (Level 3), and extreme impairment (Level 4).

It is by means of the pathological traits that the stylistic expression of personality dysfunction is characterized in both these classification systems. Importantly, the ICD-11 model does not require the presence of pathological personality traits for a PD diagnosis, although an association between severe personality dysfunction and several pathological traits may be assumed, whereas the DSM-5 model defines trait constellations that distinguish six personality disorders. However, even for the DSM-5 model, traits are secondary to the level of psychological impairment, given that the diagnostic threshold for a PD diagnosis is determined by the LPFS (i.e., moderate or greater impairment on the LPFS) and not by the traits (12, 13).

The two models take negative affectivity, detachment, antagonism/dissociality, and disinhibition (trait domains) into consideration. However, some differences may be noted as far as the psychoticism domain is concerned, which does not feature as a personality trait domain in the ICD-11 (6), and in terms of the anankastia domain, corresponding to compulsivity in the DSM-5, which was removed from the final AMPD model (7). Furthermore, facets are acknowledged by the DSM-5 within each trait domain, while this does not occur in the ICD-11.

Despite psychoticism being regarded not as a personality trait but as a feature of schizophrenic spectrum disorders by the ICD-11, it should be noted that the DSM-5 psychoticism domain captures features that range from atypical behaviors and experiences which are, nonetheless, close to normal functioning, to the more extreme manifestations of this trait domain, these indeed akin to schizophrenic-like features (7). In addition, the AMPD’s early versions consisted of a compulsivity domain, analogous to anankastia (14), which was withdrawn from the final model for parsimonious reasons, despite experts’ contention that high scores on the rigid perfectionism and perseveration facets do not reflect the complexity of compulsive/anankastic functioning (15, 16), and that a separate compulsivity/anankastia domain is supported by the research (17–19).

The central features of the PD diagnosis are operationalized in both the ICD-11 and DSM-5. In the AMPD, the LPFS was operationalized into a self-report, the LPFS-SR (20), allowing for a more objective assessment of the level of personality dysfunction, as well as more research on the LPFS construct. The Personality Inventory for the ICD-11 (PiCD) (21) and the Personality Inventory for the DSM-5 (PID-5) (22) were developed to measure the specific trait features and have been found to effectively evaluate the maladaptive traits of each model, thus facilitating clinicians’ identification of the most prominent traits in each patient.

In conceptual terms, there is a distinction between personality (dys)functioning and pathological personality traits. Notwithstanding, an empirical overlap between maladaptive personality functioning and maladaptive personality content has been identified by the research (4, 12) and this has been used to question the utility of personality impairment as operationalized by the LPFS-SR, and to defend that pathological traits alone are more useful to capture the severity of the dysfunction (23). In line with Zimmermann’s appeal (24), beyond defending or abolishing AMPD Criterion A, research should focus on how these dimensions of personality dysfunction interweave.

In accordance, recent evidence supports that the severity of personality dysfunction reflects the global quality (“g-factor”) that connects all the maladaptive personality characteristics (8, 25–30). Moreover, both the ICD-11 and DSM-5 PD classifications recognize that in more severe cases of personality dysfunction, a greater number of traits with high scores and more complex constellations of traits are observed, thus revealing the severity of their personality impairment, which in turns predicts whether they have more than one PD or one of the more typically severe PDs (6–8). Additionally, research supports the use of total and individual trait scores as a proxy of severity of the individual’s personality dysfunction (7, 27, 31).

The WHO is the authoritative mental disorders classification system and in view of the afore-mentioned similarities between the two classification systems, it would be helpful to clinical practice and the diagnostic process if these two systems were harmonized (17, 18, 32, 33), thus it has been suggested that ICD-11 PDs can be assessed using instruments based on the AMPD (34).

The PID5BF + M (17, 18), a modified version of the PID5BF + (35), is an algorithm that assesses the DSM-5 and ICD-11 six trait domains (negative affectivity, detachment, antagonism/dissociality, disinhibition, anankastia and psychoticism) and 18 facets, thus contributing to the effort of harmonizing both PD classification systems. Although personality is not described at a facet level in the ICD-11, studies with the PID5BF + M have suggested that the ICD-11 trait domain can be suitably characterized by the DSM-5 trait facets (17, 18, 26).

In addition to a study conducted by Zimmermann et al. (31), which observed the suitable alignment of the PID5BF + total score with several PD severity measures, Pires et al. (27) provided evidence on the utility of the PID5BF + M total score as a global measure of the severity of personality dysfunction, as well as on its ability to characterize specific PD trait expressions.

Pursuing the harmonization of the ICD-11 and DSM-5 PD classification systems, it was the aim of this study to offer further support to the convergent validity of the PID5BF + M through the comparison of the PID5BF + M, the LPFS-SR and the PID-5 utility in differentiating individuals with and without a PD diagnosis. Given that the PID5BF + M total can be seen as a measure of the level of personality dysfunction, strong relations were expected between the PID5BF + M total and the LPFS-SR total. Considering that the PID5BF + M is comprised of items from the PID-5, strong relations were also expected between the trait specifiers measured by the PID5BF + M and the PID-5. Moreover, differences between the community and clinical samples were expected by means of the dimensions assessed through the three afore-mentioned measures.

## Materials and methods

### Participants and procedures

The community sample was a convenience sample of the Portuguese general population, mostly from the Lisbon area, recruited from the relatives and acquaintances of undergraduate students from a higher education institution. This student-recruited sampling method can be considered a variant of the snowball sampling technique. The sample consisted of 280 participants aged between 21 and 83 years (*M*_age_ = 48.01 years, SD = 10.89 years, 46.8% males, 53.2% females), the majority of whom were employed (83.2%) and had 12 or more years of schooling (83.8%).

The sample of patients with PD consisted of 131 patients aged between 18 and 76 years (*M*_age_ = 42.66 years, SD = 12.26 years, 55.0% males, 45.0% females), 55.7% of whom had a diagnosis of borderline personality disorder. Most of the PD patients (59.5%) met the criteria for at least one comorbidity, with depressive disorders, substance-related and addictive disorders, bipolar and related disorders being the most frequent. Data collection coincided with a period during which the patients were undergoing mental health treatment and the diagnoses were attributed by the psychiatrist responsible for each patient, according to the DSM-5 classification. The exclusion criteria consisted of diagnoses of intellectual disability, schizophrenia, and major and mild neuro-cognitive disorders. The PD sample was less educated than the community sample (48.8% with 12 or more years of schooling), and mostly unemployed (60.9%).

Data collection occurred in the context of the adaptation of the LPFS-SR and the PID-5 for Portugal. Both translated versions have proven their relevance in terms of reliability, as well as validity in clinical, and non-clinical samples [see details (36, 37); LPFS-SR data is unpublished]. The research ethics committees of the host and affiliated institutions approved the study and research protocol, composed of a socio-demographic questionnaire and four personality tests, two of which were the LPFS-SR and the PID-5. Information was provided to the patients regarding their voluntary participation and the possibility of withdrawing from the study at any time. Additionally, they were ensured that they would not be asked identifying information and that the data would be used solely for the purposes of a scientific study.

### Instruments

The Modified Version of the Personality Inventory for DSM-5–Brief Form Plus (PID5BF + M) (18). The Personality Inventory for DSM-5–Brief Form Plus (PID5BF +) (35) is a self-report consisting of 34 items which was designed to merge DSM-5 and ICD-11 traits in six domains (negative affectivity, detachment, antagonism/dissociality, disinhibition, anankastia and psychoticism). Ant colony optimization algorithms were used to select the items from the original pool of PID-5 items [see details in Kerber et al. (35)]. The Modified Version of the Personality Inventory for DSM-5–Brief Form Plus (PID5BF + M) (18), involved a revision of the anankastia domain and an empirical validation of its operationalization changes [see details in Bach et al. (18)]. The PID5BF + M consists of 36 items that distinguish 18 facets in the six trait domains (three facets for each domain). The greater the dysfunction in a specific trait facet or domain the higher the score (18). In line with the procedure of Zimmermann et al. (31) for the PID5BF +, a PID5BF + M total score was also calculated as a global index of personality dysfunction. Our PID5BF + M data stemmed from full PID-5 data.

Level of Personality Functioning Scale–Self-Report (LPFS-SR) (20). The LPFS-SR is a self-report measure which operationalizes Criterion A of the DSM-5 Alternative Model of Personality Disorders, and which derives from the LPFS (7, 11), the rating scale proposed in the DSM-5 to characterize the level of personality functioning. It is composed of 80 items, rated on a 4-point Likert scale ranging from 1 (totally false, not at all true) to 4 (very true) which characterize a global severity level and four dimensions of personality dysfunction (Identity, Self-Direction, Empathy, and Intimacy). The adequate psychometric properties of the LPFS-SR guarantee its suitability for a deeper understanding of personality dysfunction and its relations with personality traits.

The Personality Inventory for DSM-5 (PID-5) (7, 22). The PID-5 self-report measure operationalizes Criterion B of the DSM-5 Alternative Model of Personality Disorders. It is comprised of 220 items characterizing 25 facets organized into five major domains of maladaptive personality variation (negative affectivity, detachment, antagonism, disinhibition, and psychoticism), and rated on a 4-point Likert scale, ranging from 0 (very false or often false) to 3 (very true or often true). The PID-5 has been studied worldwide, both in clinical and non-clinical samples, and has shown sound psychometric features such as replicable factor structure, internal consistency, convergence with personality measures, and with a broad range of psychopathological constructs.

### Data analysis

Statistical data analyses were performed with the *IBM SPSS Statistics* (Version 27). Internal consistency for the PID5BF + M, the LPFS-SR and the PID-5 scales was examined through Cronbach’s alpha coefficient. Given that in both the community and clinical samples most of the scales scores frequently revealed skewed distributions as well as problems of marked heterogeneity in an unbalanced design (in some cases, the variance ratio, dividing the value of the largest variances by the smallest variance is greater than 5), there are well-known concerns regarding the use of Pearson’s correlation and Student’s *t* test. Thus, convergent validity analyses were calculated with the Spearman correlation coefficient and the mean rank score differences between the two samples were examined by means of the Mann–Whitney U test for independent samples. Moreover, with a large *N*, effect size measures are indispensable, and some general guidelines were provided by Cohen (38) to determine the strength of a correlation: small when 0.10 ≤ *r* < 0.30, medium when 0.30 ≤ *r* < 0.50 and large when *r* ≥ 0.50. For the Mann–Whitney *U* test, effect size was evaluated through *r* = *Z*/√*N*, *N* = *n*_community_ + *n_*PD*_.*

## Results

Cronbach’s alphas for the six PID5BF + M domains were moderate and lower than those obtained for the five PID-5 domains. In the community sample, the PID5BF + M mean Cronbach’s alpha was 0.71, ranging from 0.60 at the lowest level for disinhibition to 0.75 for anankastia. In the PD sample, the mean Cronbach’s alpha was 0.69, ranging from 0.62 at the lowest level for detachment to 0.74 for psychoticism. As for the total PID5BF + M score, in the community sample the alpha was 0.88, and in the PD sample the alpha was 0.85.

Regarding the PID-5, in the community sample, the mean Cronbach’s alpha was 0.90, ranging from 0.87 at the lowest level for antagonism to 0.93 for psychoticism. In the PD sample, the mean Cronbach’s alpha was also 0.90, ranging from 0.87 at the lowest level for detachment to 0.94 for psychoticism.

Finally considering the LPFS-SR, in the community sample the mean Cronbach’s alpha was 0.78, ranging from 0.67 at the lowest level for empathy to 0.87 for identity. In the PD sample the mean Cronbach’s alpha was 0.75, ranging from 0.49 at the lowest level for empathy to 0.87 for self-direction. As for the LPFS-SR total score, in both samples the alpha was 0.94.

Table 1 presents Spearman’s correlations between the PID5BF + M total and LPFS-SR total and domains in the community sample and personality disorder (PD) samples. A master correlation matrix between the PID5BF + M domains and facets and LPFS-SR total and domains in both samples is presented as a Supplementary Appendix.

**TABLE 1:** Spearman’s correlations between the PID5BF + M total and LPFS-SR total and domains in the community sample and personality disorder (PD) samples.

	PID5BF + M total	
	**Community sample**	**PD sample**
LPFS-SR total	0.59**	0.84**
Identity	0.61**	0.67**
Self-direction	0.48**	0.66**
Empathy	0.50**	0.60**
Intimacy	0.43**	0.79**

**p < 0.01; small correlation: 0.10 ≤ *r* < 0.30, medium correlation: 0.30 ≤ *r* < 0.50, large correlation: *r* ≥ 0.50.

Spearman’s correlations between the PID5BF + M and the PID-5 in the community sample and personality disorder (PD) samples are displayed in Table 2.

**TABLE 2:** Spearman’s correlations between the PID5BF + M and the PID-5 in the community sample and personality disorder (PD) samples.

PID5BF + M	PID5 counterparts	
	**Community sample**	**PD sample**
Emotional lability	0.87**	0.86**
Anxiety	0.86**	0.82**
Separation insecurity	0.86**	0.81**
Withdrawal	0.80**	0.78**
Anhedonia	0.75**	0.75**
Intimacy avoidance	0.69**	0.81**
Manipulativeness	0.61**	0.80**
Deceitfulness	0.80**	0.78**
Grandiosity	0.74**	0.81**
Irresponsibility	0.75**	0.78**
Impulsivity	0.42**	0.87**
Distractibility	0.85**	0.74**
Unusual beliefs and exp.	0.73**	0.74**
Eccentricity	0.79**	0.85**
Perceptual dysregulation	0.62**	0.67**
Negative affectivity	0.91**	0.89**
Detachment	0.87**	0.86**
Antagonism	0.84**	0.91**
Disinhibition	0.81**	0.89**
Psychoticism	0.84**	0.90**

**p < 0.01; small correlation: 0.10 ≤ *r* < 0.30, medium correlation: 0.30 ≤ *r* < 0.50, large correlation: *r* ≥ 0.50; Unusual beliefs and exp = Unusual beliefs and experience.

The correlations between the PID5BF + M total and LPFS-SR total and domains were statistically significant (*p* < 0.01) in both samples, and large, apart from Self-Direction and Intimacy in the community sample, in which they were medium (0.49 and 0.41, respectively). The correlations were stronger in the PD sample, particularly between the PID5BF + M total and LPFS-SR total. The correlations between the PID5BF + M facets and domains and their PID-5 counterparts were also statistically significant (*p* < 0.01) and large, apart from Impulsivity in the community sample (0.42). Again, these correlations were stronger mostly in the PD sample.

Table 3 presents the mean ranks, the independent samples Mann–Whitney U test and effect sizes in the community sample and PD samples for the PID5BF + M and the PID-5. The PD group showed significantly higher scores in all traits (*p* < 0.001), thus both tests were able to differentiate clinical from non-clinical personality functioning, although the PID-5 revealed larger effect sizes.

**TABLE 3:** PID5BF + M and PID5: Mean ranks, independent samples Mann–Whitney U test (*Z*), and effect sizes (*r*) in the community sample (C) and personality disorder (PD) samples.

Scales	Samples	PID5BF + M				PID5			
		**Mean ranks**	***Z***	***P***	***r***	**Mean ranks**	***Z***	***P***	***r***
Emotional lability	C	174.69	-8.18	<0.001	0.40	164.96	-10.26	<0.001	0.50
	PD	276.56				293.71			
Anxiety	C	172.84	-8.64	<0.001	0.43	164.55	-10.35	<0.001	0.51
	PD	280.53				294.60			
Separation insecurity	C	176.65	-7.72	<0.001	0.38	169.44	-9.14	<0.001	0.45
	PD	272.34				284.15			
Withdrawal	C	172.55	-9.13	<0.001	0.45	170.17	-8.96	<0.001	0.44
	PD	281.17				282.59			
Anhedonia	C	167.87	-10.19	<0.001	0.50	168.60	-9.35	<0.001	0.46
	PD	291.24				285.95			
Intimacy avoidance	C	180.02	-10.19	<0.001	0.50	177.92	-9.35	<0.001	0.46
	PD	265.07				266.02			
Manipulativeness	C	183.41	-7.07	<0.001	0.35	179.14	-6.63	<0.001	0.33
	PD	257.78				261.65			
Deceitfulness	C	175.46	-8.16	<0.001	0.40	173.91	-8.06	<0.001	0.40
	PD	274.89				274.59			
Grandiosity	C	188.67	-4.91	<0.001	0.24	183.51	-5.65	<0.001	0.28
	PD	246.45				254.07			
Irresponsibility	C	184.77	-6.04	<0.001	0.30	165.65	-10.22	<0.001	0.50
	PD	254.86				292.24			
Impulsivity	C	187.27	-5.05	<0.001	0.25	161.77	-11.07	<0.001	0.54
	PD	249.48				300.54			
Distractibility	C	174.06	-8.36	<0.001	0.41	164.27	-10.43	<0.001	0.51
	PD	277.91				295.19			
U beliefs and exp.	C	171.41	-9.40	<0.001	0.46	165.41	-10.20	<0.001	0.50
	PD	283.62				292.75			
Eccentricity	C	172.53	-9.26	<0.001	0.46	160.56	-11.40	<0.001	0.56
	PD	281.21				303.12			
Perceptual dysr.	C	183.95	-6.43	<0.001	0.32	160.28	-11.44	<0.001	0.56
	PD	256.61				303.72			
Perfectionism	C	186.31	-5.25	<0.001	0.26				
	PD	251.54							
Rigidity	C	188.80	-4.63	<0.001	0.23				
	PD	246.17							
Orderliness	C	187.85	-4.93	<0.001	0.24				
	PD	248.23							
Attention seeking	C					171.04	-8.76	<0.001	0.43
	PD					280.73			
Callousness	C					168.27	-9.46	<0.001	0.47
	PD					286.64			
Depressivity	C					154.41	-12.91	<0.001	0.64
	PD					316.27			
Hostility	C					170.19	-8.95	<0.001	0.44
	PD					282.55			
Perseveration	C					161.56	-11.12	<0.001	0.55
	PD					301.00			
Restricted affectivity	C					178.25	-6.95	<0.001	0.34
	PD					265.32			
Rigid perfectionism	C					178.64	-6.83	<0.001	0.34
	PD					264.48			
Risk taking	C					179.40	-6.64	<0.001	0.33
	PD					262.86			
Submissiveness	C					172.36	-8.49	<0.001	0.42
	PD					277.91			
Suspiciousness	C					168.32	-9.43	<0.001	0.46
	PD					286.55			
Negative affectivity	C	165.96	-10.28	<0.001	0.51	159.45	-11.61	<0.001	0.57
	PD	295.34				305.49			
Detachment	C	163.08	-11.11	<0.001	0.55	164.88	-10.26	<0.001	0.50
	PD	301.55				293.88			
Antagonism	C	174.00	-8.33	<0.001	0.41	174.84	-7.65	<0.001	0.38
	PD	278.05				270.79			
Disinhibition	C	173.10	-8.50	<0.001	0.42	157.94	-11.99	<0.001	0.59
	PD	279.97				308.73			
Psychoticism	C	165.83	-10.43	<0.001	0.51	158.21	-11.92	<0.001	0.59
	PD	295.63				308.14			
Anankastia	C	182.38	-6.17	<0.001	0.30				
	PD	259.99							
Total PID5BF + M	C	158.39	-12.15	<0.001	0.60				
	PD	311.65							

*r* = Z/$\sqrt{N}$, *N* = *n*_community_ + *n_PD_*; small effect: 0.10 ≤ *r* < 0.30, medium effect: 0.30 ≤ *r* < 0.50, large effect: *r* ≥ 0.50. U beliefs & exp. = Unusual beliefs & experience; Perceptual dysr. = Perceptual dysregulation.

The mean ranks, the independent samples Mann–Whitney U test and effect sizes in the community sample and PD samples for the LPFS-SR are displayed in Table 4. The PD group also showed significantly higher scores in all the LPFS-SR domains (*p* < 0.001), thus confirming that the LPFS-SR is also able to statistically differentiate clinical from non-clinical personality functioning.

**TABLE 4:** LPFS-SR: Mean ranks, independent samples Mann–Whitney U test (*Z*), and effect sizes (*r*) in the community sample (C) and personality disorder (PD) samples.

LPFS-SR					
**Scales**	**Samples**	**Mean ranks**	***Z***	***P***	***r***
Identity	C	142.75	−7.23	<0.001	0.36
	PD	255.66			
Self-direction	C	143.99	−7.00	<0.001	0.34
	PD	252.77			
Empathy	C	144.28	−5.90	<0.001	0.29
	PD	235.83			
Intimacy	C	141.08	−6.56	<0.001	0.32
	PD	244.97			
Total	C	129.51	−6.61	<0.001	0.33
	PD	230.39			

*r* = Z/$\sqrt{N}$, *N* = *n*_community_ + *n_PD_*; small effect: 0.10 ≤ *r* < 0.30, medium effect: 0.30 ≤ *r* < 0.50, large effect: *r* ≥ 0.50.

## Discussion

The aim of this study was to contribute to the convergent validity of the PID5BF + M, a test that bridges both the ICD-11 and the DSM-5 PD classification systems, through the study of its ability to differentiate patients with a PD diagnosis from a community sample, in comparison with the LPFS-SR and the PID-5, that assess the level of personality dysfunction and specific personality traits, respectively.

Although the PID5BF + M assesses trait qualifiers and not the level of PD severity *per se*, research supports the use of total and individual trait scores as a proxy of severity of the individual’s personality dysfunction (7, 27, 31). This finding is in line with the conceptualization of the ICD-11, namely, that the number, complexity and permeative nature of pathological traits may indicate severity (39), and with the idea that severity may be measured by the AMPD trait model by adding the number of pathological traits presented (40). Furthermore, the global quality (“g-factor”) that links all the maladaptive personality features has been found to be represented by severity (25, 28–30). Therefore, it stands to reason that maladaptive traits are likely to be more prominent in those with more severe personality dysfunction (i.e., personality disorders).

Thus, in this study, the PID5BF + M total score along with the specific domain and facet scores were expected to differentiate personality disordered patients from a community sample. Moreover, if it may be reasonably predicted that those with more severe personality dysfunction should have more, and more complex, pathological personality traits, the sum of pathological traits presented (PID5BF + M total) should relate strongly to the LPFS-SR, the test that assesses the level of personality dysfunction in the AMPD. Furthermore, the PID5BF + M pathological traits themselves should relate strongly to their PID-5 counterparts.

The internal consistency of the PID5BF + M six domains and total score, in the community and PD samples, was addressed and compared with the LPFS-SR and PID-5 internal consistencies. Considering the PID5BF + M domains, the mean Cronbach’s alphas were lower than those obtained for the PID-5 and ranged from 0.69 in the PD sample to 0.71 in the community sample. The few items per domain (domains consisting of three facets with two items each) may have given rise to these moderate alphas, which are in line with the mean domain reliability obtained in a recent study with a non-clinical sample (41). However, the PID5BF + M total score showed high internal consistency in both samples, 0.85 in the PD sample and 0.88 in the non-PD sample. Moreover, these results were in keeping with those obtained for the LPFS-SR total, 0.94 in both samples, thus revealing the reliability of both the PID5BF + M total and the LPFS-SR total in addressing personality dysfunction.

Considering the correlations between the PID5BF + M total and the LPFS-SR scales, and in accordance with our expectations, significant and medium to large correlations were found in all scales, stronger in the PD sample and specifically between the PID5BF + M total and the LPFS-SR total. Therefore, our results suggest that the PID5BF + M total relates strongly to a measure of the severity of personality dysfunction, thus confirming previous research (27, 31) and contributing to its convergent validity.

The fact that the strongest relations were observed between the LPFS-SR total and PID5BF + M total corroborate the evidence that regards the LPFS-SR construct as a single dimension of personality pathology, and questions its ability to capture deficits in identity, self-direction, empathy, and intimacy (20, 42–46). Considering that these results appear to support the LPFS-SR’s one-dimensionality, a controversial issue for Criterion A’s opponents (23), they may also offer a modest contribution to the constructive discussion regarding the strengths and limitations of Criterion A (24).

Regarding the PID5BF + M’s convergent validity with the PID-5, in the community sample, the mean correlations for the facets and domains were 0.74 and 0.85, respectively. In the PD sample, the mean correlations for the facets and domains were 0.79 and 0.89, respectively. The fact that the correlations between the PID5BF + M scales and their PID-5 counterparts were all statistically significant and large, apart from Impulsivity in the community sample, is in line with Riegel et al.’s (41) findings and suggests that the PID5BF + M is as useful as the PID-5 in capturing the stylistic features of personality disorders, thus sustaining its validity. Despite the PID5BF + M being comprised of items from the PID-5, in this new measure solely two items per facet were retained. Thus, although significant correlations between the PID5BF + M and the PID-5 were not totally unexpected, small differences in the functioning of an item may affect correlations and this may have accounted for the Impulsivity correlation in the community sample.

Finally, regarding group differences, all the scales in the three tests compared (PID5BF + M, LPFS-SR, PID-5) revealed higher means in the PD sample, despite the tendency for PID-5 differences to reveal slightly higher effect sizes in comparison to the PID5BF + M and the LPFS-SR. Moreover, our results showed a greater association of the PID5BF + M total score with the clinical group status compared to the association of the LPFS-SR total score with the clinical group status, which suggests that the PID5BF + M has potential for the assessment of the severity of personality dysfunction.

Several studies have offered support for the PID-5 [see for a review (47)] and the LPFS-SR’s (20, 44) discriminative capacity between clinical and non-clinical samples. The results of the current study, contribute to the empirical validity of the PID5BF + M, confirming its utility in differentiating clinical from non-clinical participants, thus supporting its use as a valid alternative instrument for assessing personality pathology.

In a nutshell, this comparative study shows the consistency between the PID5BF + M scores and the LPFS-SR and PID-5 scores and thus corroborates the ability of the PID5BF + M to mirror the severity of personality dysfunction (27, 31), and to depict the specific traits underlying the stylistic manifestations of PD (17, 18, 27).

Of note, the prominent relations found between the PID5BF + M total and the LPFS-SR total are in keeping with recent studies pointing to the secondary role of trait domains in the identification of general PD (12, 48), which in turn appears to support the ICD-11 model for which pathological personality traits do not need to be present for a PD diagnosis (6), and the DSM-5 model where the LPFS and not the pathological trait model determines the diagnostic threshold for a PD diagnosis (7).

Given that our PID5BF + M data was extracted from the original 220-item PID-5, the relations found between the PID5BF + M scales and their PID-5 counterparts were not unexpected, however they may not be attributed solely to the dataset given that Riegel et al. (41) recently proved the invariance of an extracted version of the test and its standalone version. Therefore, considering the length of the original PID-5, and despite the somewhat smaller effect sizes in the PID5BF + M group differences, mostly in research settings but also in clinical contexts, it might be preferable to use a smaller reliable instrument, with proven convergent validity, which our results appear to attest.

The study’s limitations should also be borne in mind when considering the findings. The heterogeneity in the level of schooling of both samples, which may not have ensured appropriate comparability of the clinical and non-clinical samples, the large number of comorbid disorders within the PD sample, the small number of patients and the fact that no direct screening for psychopathology in the community sample had been performed are some of the most relevant. In addition, this study is based exclusively on a self-report methodology and socially desirable responding bias or denial/defensiveness have been widely referred to as factors that affect the accuracy of self-reports for research purposes, particularly when questions are related to sensitive topics such as emotions, cognitions, and behaviors (49–51). Therefore, it should be noted that not having relied on multi-method and/or clinician assessment of PD severity and trait/style is one of the major limitations of this study, which should be corrected in future research to improve its validity. However, our results corroborate the use of the PID5BF + M for PD assessment in view of its potential to identify more severe personality dysfunction cases, i.e., individuals with higher scores in most of the maladaptive traits. Additionally, its ability to highlight the specific stylistic expressions of the personality dysfunction should be noted. Furthermore, as the PID5BF + M traits comprehend the traits described in the ICD-11 PD classification system and the DSM-5 AMPD, this study, along with prior research (18, 27, 31, 41), may contribute to developments of the DSM-5 in future studies, namely, by bringing it closer to the ICD-11, the mental disorders classification system established by the WHO. Finally, despite acknowledging that personality assessment is an integrative process in which the clinician brings together clinical judgment, theory and data from multiple sources (i.e., clinical observations, observant-reports, self-reports, projective techniques) in an effort to understand the person and his/her behavior (52), this study has advantages for clinical practice and for the diagnosis of PD as it shows that with a single, relatively small instrument, it is possible to cover both dimensions of the PD diagnosis, namely, the severity of personality dysfunction and its stylistic features.

## Data availability statement

The raw data supporting the conclusions of this article will be made available by the authors, without undue reservation.

## Ethics statement

The studies involving human participants were reviewed and approved by Comissão de Ética da Faculdade de Psicologia da Universidade de Lisboa; Comissão de Ética do Centro Académico de Medicina de Lisboa (CHLN/FMUL/IMM); Comissão de Ética para a Saúde do Centro Hospitalar de Lisboa Ocidental; Comissão de Ética para a Saúde do Centro Hospitalar Psiquiátrico de Lisboa; Comissão de Ética para a Saúde do Centro Hospitalar Barreiro Montijo. The patients/participants provided their written informed consent to participate in this study.

## Author contributions

RP designed the study, provided the “State of the art” revision, supervised the research project and data collection, performed the data analysis and its interpretation, and wrote the first draft of the manuscript. JH-C collaborated in the study design, supervised the research project and data collection, and provided the critical revisions. ASF performed and supervised the data analysis and interpretation. JGM, ARM, BCB, and MP supervised the data collection and provided the critical revisions. BG collaborated in the study design, coordinated the research project and data collection, and provided critical revisions. All authors contributed to the article and approved the submitted version.
