# Supplementary material for: Bridging the ICD11 and the DSM-5 personality disorders classification systems: The role of the PID5BF + M
Source: Front Psychiatry. 2023 Mar 2;14:1004895. doi: 10.3389/fpsyt.2023.1004895 (PMC10017429; doi:10.3389/fpsyt.2023.1004895)
Supplement: Supplementary file 1 [file Table_1.pdf]

Supplemental Appendix

Appendix A

Spearman's correlations between the PID5BF+M domains and facets and the LPFS-SR total and domains in the community sample and personality disorder (PD) samples

PID5BF+M	LPFS-SR									
	Community Sample					PD Sample				
	Total	Ident	Self	Emp	Intim	Total	Ident	Self	Emp	Intim
Emotional lability	.34**	.40**	.32**	.28**	.21**	.60**	.58**	.38*	.27	.58**
Anxiety	.39**	.41**	.29**	.32**	.29**	.59**	.63**	.41*	.32	.64**
Sep insecurity	.35**	.38**	.29**	.30**	.24**	.38*	.44**	.38*	.31	.34*
Withdrawal	.46**	.42**	.38**	.36**	.43**	.67**	.56**	.39*	.35*	.74**
Anhedonia	.47**	.46**	.44**	.37**	.37**	.48**	.40*	.64**	.23	.39*
Intim avoidance	.40**	.37**	.34**	.32**	.34**	.03	.07	-.11	-.09	.13
Manipulativeness	.26**	.31**	.25**	.21**	.18**	.15	.05	.32*	.10	.08
Deceitfulness	.31**	.32**	.28**	.19**	.25**	.30	.25	.12	.40*	.17
Grandiosity	.26**	.25**	.20**	.26**	.24**	.40*	.24	.23	.49**	.21
Irresponsibility	.36**	.39**	.35**	.28**	.20**	.43*	.06	.37*	.42**	.37*
Impulsivity	.24**	.19**	.21**	.17**	.16**	.61**	.44**	.55**	.47**	.35*
Distractibility	.37**	.46**	.39**	.28**	.18**	.59**	.52**	.46**	.37*	.46**
U beliefs & exp.	.26**	.27**	.25**	.27**	.18**	.52**	.48**	.51**	.35*	.45**
Eccentricity	.31**	.36**	.23**	.32**	.21**	.63**	.41*	.43**	.50**	.64**
Perceptual dysr	.26**	.28**	.28**	.21**	.13*	.38*	.30	.46**	.41*	.09
Perfectionism	.24**	.23**	.18**	.28**	.14*	.36*	.26	.25	.35*	.42*
Rigidity	.15*	.16**	.00	.14*	.15*	.23	.15	-.08	.02	.39*

Orderliness	.18**	.19**	.08	.25**	.19**	.16	.16	.03	.22	.12
Neg affectivity	.47**	.52**	.39**	.39**	.32**	.63**	.65**	.47**	.37*	.60**
Detachment	.58**	.55**	.51**	.46**	.49**	.60**	.55**	.46**	.24	.65**
Antagonism	.39**	.39**	.35**	.29**	.32**	.44*	.26	.28	.48**	.26
Disinhibition	.46**	.50**	.44**	.34**	.26**	.71**	.48**	.63**	.53**	.52**
Psychoticism	.36**	.38**	.32**	.37**	.23**	.65**	.50**	.59**	.54**	.54**
Anankastia	.26**	.27**	.13*	.30**	.22**	.32	.25	.10	.24	.40*

Note. ** $p < .01$; * $p < .05$; Ident = Identity; Self = Self-Direction; Emp = Empathy; Sep insecurity = Separation insecurity; Intim = Intimacy; Intim avoidance = Intimacy avoidance; U beliefs & exp = Unusual beliefs & experience; Perceptual dysr = Perceptual dysregulation; Neg affectivity = Negative affectivity
